# Assessment of the Diagnostic Ability of Four Detection Methods Using Three Sample Types of COVID-19 Patients

**DOI:** 10.3389/fcimb.2021.685640

**Published:** 2021-06-07

**Authors:** Fei Yu, Guoliang Xie, Shufa Zheng, Dongsheng Han, Jiaqi Bao, Dan Zhang, Baihuan Feng, Qi Wang, Qianda Zou, Ruonan Wang, Xianzhi Yang, Weizhen Chen, Bin Lou, Yu Chen

**Affiliations:** ^1^ Department of Laboratory Medicine, The First Affiliated Hospital, Zhejiang University School of Medicine, Hangzhou, China; ^2^ Key Laboratory of Clinical In Vitro Diagnostic Techniques of Zhejiang Province, Hangzhou, China; ^3^ Institute of Laboratory Medicine, Zhejiang University, Hangzhou, China; ^4^ State Key Laboratory for Diagnosis and Treatment of Infectious Diseases, National Clinical Research Center for Infectious Diseases, Collaborative Innovation Center for Diagnosis and Treatment of Infectious Diseases, The First Affiliated Hospital, Zhejiang University School of Medicine, Hangzhou, China

**Keywords:** COVID-19, SARS-CoV-2, sample type, saliva, dd-RT-PCR, RT-RAA

## Abstract

**Background:**

Viral nucleic acid detection is considered the gold standard for the diagnosis of coronavirus disease 2019 (COVID-19), which is caused by SARS-CoV-2 infection. However, unsuitable sample types and laboratory detection kits/methods lead to misdiagnosis, which delays the prevention and control of the pandemic.

**Methods:**

We compared four nucleic acid detection methods [two kinds of reverse transcription polymerase chain reactions (RT-PCR A: ORF1ab and N testing; RT-PCRB: only ORF1ab testing), reverse transcription recombinase aided amplification (RT-RAA) and droplet digital RT-PCR (dd-RT-PCR)] using 404 samples of 72 hospitalized COVID-19 patients, including oropharyngeal swab (OPS), nasopharyngeal swabs (NPS) and saliva after deep cough, to evaluate the best sample type and method for SARS-CoV-2 detection.

**Results:**

Among the four methods, dd-RT-PCR exhibited the highest positivity rate (93.0%), followed by RT-PCR B (91.2%) and RT-RAA (91.2%), while the positivity rate of RT-PCR A was only 71.9%. The viral load in OPS [24.90 copies/test (IQR 15.58-129.85)] was significantly lower than that in saliva [292.30 copies/test (IQR 20.20-8628.55)] and NPS [274.40 copies/test (IQR 33.10-2836.45)]. In addition, if OPS samples were tested alone by RT-PCR A, only 21.4% of the COVID-19 patients would be considered positive. The accuracy of all methods reached nearly 100% when saliva and NPS samples from the same patient were tested simultaneously.

**Conclusions:**

SARS-CoV-2 nucleic acid detection methods should be fully evaluated before use. High-positivity rate methods such as RT-RAA and dd-RT-PCR should be considered when possible. Furthermore, saliva after deep cough and NPS can greatly improve the accuracy of the diagnosis, and testing OPS alone is not recommended.

## Introduction

Since the first emerging in late 2019, coronavirus disease 2019 (COVID-19) has caused a worldwide pandemic, with more than 157 million confirmed cases and 3 million deaths (WHO, 2021). Early diagnosis and treatment of suspected patients is the key to effectively control COVID-19 (Chu et al., 2020; Li et al., 2020). Currently, viral nucleic acid detection is still the most effective method to confirm SARS-CoV-2 infection. A variety of detection methods based on specific SARS-CoV-2 nucleotide sequences have been rapidly developed and used as emergency applications in the laboratory. To date, National Medical Products Administration (NMPA China) has approved 22 SARS-CoV-2 nucleic acid detection reagents, most of which are reverse transcription polymerase chain reaction (RT-PCR) methods. In addition, some other detection techniques are waiting for approval, such as reverse transcription recombinase aided amplification (RT-RAA) method and droplet digital RT-PCR (dd-RT-PCR) method. RAA is a new type of nucleic acid amplification technology developed in recent years, and it works using four enzymes (UvsX, UvsY, SSB, and polymerase) at a constant temperature of 37~ 42°C (Zhang et al., 2017). Compared to RT-PCR, RT-RAA based assay is faster, simpler and no need for fluorescent quantitative PCR instruments (Wang J. et al., 2020). The dd-RT-PCR is a new method of digital PCR that enables the absolute quantification of nucleic acid without the use of calibration curves (Hindson et al., 2011). Meanwhile, it has been reported that the positivity rate or sensitivity of initial RT-PCR result is compared to result after repeated tests of RT-PCR as reference standard methods, with a range of 51.25% to 94.6% (Axell-House et al., 2020). Reasons for the false negatives of initial RT-PCR may include insensitive nucleic acid detection kits, variations in the accuracies of different tests, low initial viral load or improper clinical sampling (Fan et al., 2020; Fang et al., 2020). Thus, multiple samples and repeated testing may be required to diagnose patients who are infected with SARS-CoV-2.

In this study, we compared two RT-PCR reagents approved by the NMPA China, an RT-RAA reagent, and a dd-RT-PCR reagent, using multiple types of sample of hospitalized COVID-19 patients, to evaluate the performance of different methods. Simultaneously, we compared the positivity rate of using three common sample types, namely oropharyngeal swab (OPS), nasopharyngeal swabs (NPS) and saliva after deep cough, to provide empirical evidence for the selection of suitable samples and methods in the diagnosis of SARS-CoV-2 infection.

## Materials and Methods

### Subjects

This study enrolled a total of 72 COVID-19 patients, who were admitted to the First Affiliated Hospital, Zhejiang University School of Medicine, from 19^th^ Jan 2020 to 23^rd^ Feb 2020. All enrolled cases were confirmed to be infected by SARS-CoV-2 through multiple repetitions of RT-PCR detection (RT-PCR A: BioGerm, Shanghai, China). COVID-19 patients were diagnosed according to the 6^th^ edition of the Guideline for Diagnosis and Treatment of SARS-CoV-2 issued by the National Health Commission of the People’s Republic of China. The demographic information, medical comorbidities, date of symptom onset, symptoms and signs, progression and resolution of clinical illness during the hospitalization period were obtained from the clinical records. All data were reviewed by a trained team of physicians. This study was reviewed and approved by the Clinical Research Ethics Committee of the First Affiliated Hospital, Zhejiang University School of Medicine.

### Sample Collection

Three high-concentration positive clinical samples were 5-fold serially diluted to 1: 78,125 using normal saline to evaluate the limit of detection of each method, a total of 24 samples were obtained.

Excluding those 4 patients with tracheal intubation or coma, 68 hospitalized COVID-19 patients were included in study on 11^th^ Feb. These patients were all in the general isolation wards at the time of sampling. Before sampling, it was confirmed that the patient did not drink water, eat food, gargle or other similar behaviors within half an hour that might affect the sampling quality. The specific collection process is as follows: OPS were collected first, NPS was collected after a 15-minite’ interval, and after another 15-minutes the patient was instructed to wear a mask and deep cough 3~5 times before spitting saliva into a sterile container. OPS and NPS of each patient were obtained by experienced physicians using flocked swabs (FLOQSwabs, Copan Italia) and were transported in 3 mL universal transport medium (UTM, Copan Italia). Swabs were rotated and stayed for enough time to collect the fluid and epithelial cells. All samples were sent to the laboratory for testing within 1h after sampling. A total of 204 samples were collected from the 68 patients.

**Figure 1:**
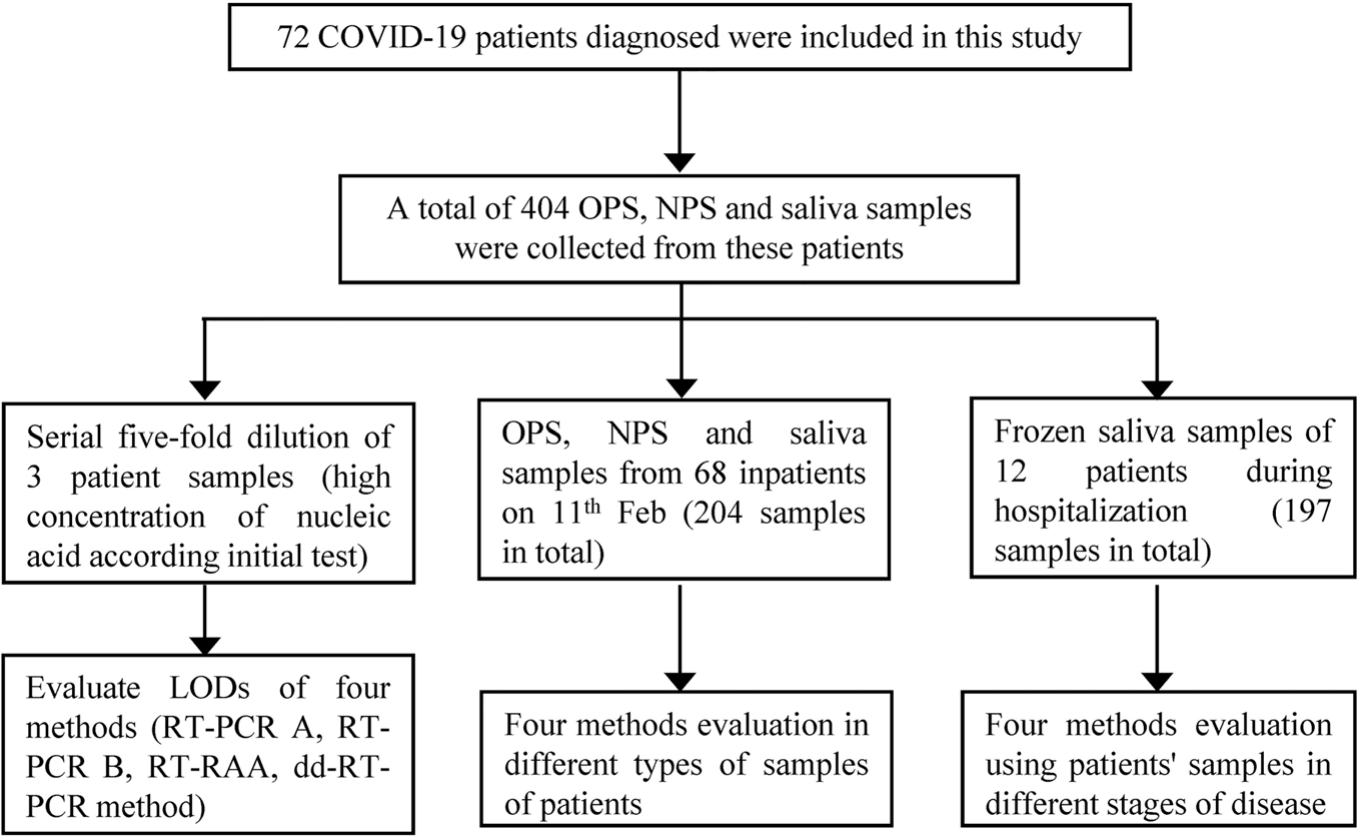
Flowchart of patient/sample recruitment and study flow.

Additionally, to dynamically evaluate four methods in different stages of disease, 12 of the 72 patients having well-preserved saliva after deep cough were enrolled in this study. A total of 197 saliva samples from these 12 hospitalized patients were collected. Each patient provided 11 to 26 saliva samples. All samples were tested on the day of collection and were frozen at -80°C after testing. The protocols of sample inclusion and testing are shown in **Figures 1** and **3**.

### Laboratory Testing

For viscous saliva samples, an equal volume of 0.4 mg/mL protease K buffer was added to the samples before nucleic acid extraction. After that, the mixture was vortexed for 15s, left standing for 20min, vortexed for another 15s, and centrifuged at 13000*rpm* for 5min. Then supernatant was collected for viral RNA extraction. Viral RNAs of saliva supernatant, OPS and NPS were extracted using the MagNA Pure LC 2.0 (Roche, Basel, Switzerland). Finally, four methods were used to detect nucleic acid of SARS-CoV-2, including two RT-PCR kits (A: Cat. No. ZC-HX-201-2, BioGerm, Shanghai, China; B: Cat. No. MFG030010, BGI Genomics, Wuhan, China), an RT-RAA reagent (Cat. No. T00R01; Qitian, Wuxi, China), and a dd-RT-PCR reagent (Cat. No. 13444; TargetingOne, Beijing, China). The principles, targets, and result interpretation of the four methods are shown in **Table 1**. Operation and result assessment were conducted in accordance with the manufacturers’ instructions. Negative and positive controls were setted for each test. In accordance with the guidelines of the Chinese Health Commission, all samples tested in this study were conducted in biosafety Level 2 laboratory. In addition, serially diluted samples were tested at the same time with three replicates, while the samples with suspected results were not tested again. The results were determined to be positive only when all three replicates were positive.

**Table 1:** Introduction of the 4 methods.

Method	RT-PCR A	RT-PCR B	RT-RAA	dd-RT-PCR
Reaction principle	Quantitative reverse transcription real-time PCR	Quantitative reverse transcription real-time PCR	Reverse transcription recombinase- aided amplification	Droplet digital reverse transcription PCR
Targets	ORF1ab and N	ORF1ab	ORF1ab	ORF1ab and N
Internal control	Yes	Yes	No	Yes
RNA load (μL)	5	10	5	15
Limit of detection (copies/mL)	1000	100	200	200
Time spent (min)	~87	~95	~17	~160
Result interpretation	Positive: Ct ≤ 38; Negative: Ct>38; Retest: single gene Ct>38. After retesting, double genes Ct ≤ 38 for positive, otherwise negative.	Positive: Ct ≤ 38; Negative: No Ct; Retest: Ct>38. After retesting, the sigmoidal curve e result is considered positive.	According to the manufacturer’s instructions, set the slope as 20 to automatically judge the test results.	Positive: Copies of ORF1ab≥3 and ORF1ab+N≥5, or N≥5; Negative: ORF1ab<3, and ORF1ab+N<5; Retest: ORF1ab<3, and ORF1ab+N≥5; After retesting, the suspicious result is considered positive. The final viral RNA copy number was defined as the higher value of the two genes.

### Statistical Analysis

Descriptive statistics included the mean with standard deviation (SD; for data with normal distribution), median with interquartile range (IQR; for data with skewed distribution), and proportion (%). If any of the four methods showed a positive result for a sample, the specimen was classified as positive, and the positivity rate of each method was calculated. The Kruskal-Wallis test was used to evaluate the viral load among different sample types detected by dd-RT-PCR. A p value less than 0.05 (two-sided) was considered statistically significant. The statistical analysis was performed using either Prism 7.0 (GraphPad, La Jolla, CA, USA) or SPSS 17.0 (College Station, TX, USA) software.

## Results

### Patient Description

The median age of the 72 patients in the study was 56 years (IQR 40-65years) and 62.5% of them were males. Fever (83.3%), cough (54.2%), and expectoration (30.6%) were the most common clinical manifestations at the time of admission. 16 patients were admitted to the ICU, and 4 of them were under mechanical ventilation. 88.9% of patients received oxygen supplement. The demographic and clinical characteristics of the cross-section group (n=68) and longitudinal group (n=12) are shown in **Table 2**. Additionally, the median of days after symptoms onset of 68 patients in the cross-section group was 15 days (IQR, 11~19 days).

**Table 2:** Demographics and clinical characteristics of enrolled patients.

Variables	No. of patients (% of total)		
	Total (N=72)	Cross-section group^a^ (N=68)	Longitudinal group^b^ (N=12)
**Demographics**			
Median age(median [IQR]) (yr)	56 (40-65)	54 (40-64)	55(39-65)
Male sex	45 (62.5)	41 (60.3)	8 (66.7)
**Underlying disease**			
Hypertension	25 (34.7)	22 (32.4)	6 (50.0)
Chronic heart disease	3 (4.2)	3 (4.4)	0 (0)
Chronic lung disease	6 (8.3)	6 (8.8)	2 (16.7)
Chronic liver disease	3 (4.2)	3 (4.4)	1 (8.3)
Diabetes	2 (2.8)	0 (0)	2 (16.7)
Solid tumor	1 (1.4)	0 (0)	1 (8.3)
**Symptoms**			
Fever	60 (83.3)	56 (82.4)	10 (83.3)
Cough	39 (54.2)	36(52.9)	7(58.3)
Sputum	22 (30.6)	19(27.9)	6(50.0)
Chest distress	7 (9.7)	7(10.3)	0(0)
Dizziness	5 (6.9)	4(5.9)	1(8.3)
Headache	3 (4.2)	3(4.4)	1(8.3)
Diarrhea	8 (11.1)	8(11.8)	1(8.3)
Myalgia	14 (19.4)	12(17.7)	4(33.3)
**Disease severity**			
Oxygen supplement	64 (88.9)	60(88.2)	12(100)
Invasive mechanical ventilation	2 (2.8)	2(2.9)	2(16.7)
Intensive care unit admission	16 (22.2)	12(17.7)	5(41.7)

^a^Simultaneously collected OPS, NPS, and saliva samples from each hospitalized patient. Due to coma during sampling, 4 of the 72 patients were excluded;

^b^12 of the 72 hospitalized patients having well-preserved saliva after deep cough were enrolled in this study.

### Performance of the 4 Methods in Diluted Positive Samples

The results showed that the performance of the four methods were different (**Table 3**). Specifically, in the testing of NPS, all four methods performed well with a dilution of 625-fold. In the 3,125-fold diluted OPS and saliva sample, the performance of RT-PCR A was not as good as those of the other three methods.

**Table 3:** Detection of SARS-CoV-2 in serial five-fold dilution of 3 samples.

Sample	Dilution	RT-PCR A		RT-PCR B ORF1ab (Ct)	RT-RAA ORF1ab (Min)	dd-RT-PCR ORF1ab and N (copies/test)^c^
		ORF1ab (Ct)	N (Ct)			
OPS	1×	26.69	27.01	25.51	0.00	1476.8
	5×	28.84	29.24	28.28	0.00	480.7
	25×	31.50	32.37	31.01	0.22	68.0
	125×	**33.71**	**35.20**	33.21	0.89	9.3
	625×	33.76**^a^**	34.98**^a^**	**36.90**	**2.89**	**3.3**
	3,125×	N	35.18**^b^**	38.19**^a^**	4.00**^b^**	0.9**^a^**
	15,625×	N	N	N	N	N
	78,125×	N	N	N	N	N
NPS	1×	27.29	28.04	26.71	0.00	12044.9
	5×	28.83	29.55	28.93	0.00	585.6
	25×	31.51	31.88	31.95	0.00	131.1
	125×	33.37	33.82	33.41	0.33	29.3
	625×	**36.87**	**36.60**	**35.97**	**1.22**	**8.9**
	3,125×	38.50**^b^**	N	38.84**^b^**	N	2.0
	15,625×	N	N	N	N	N
	78,125×	N	N	N	N	N
Saliva	1×	24.27	24.69	23.64	0.00	30921.9
	5×	25.70	26.59	25.94	0.00	4805.3
	25×	26.72	27.83	27.36	0.00	1386.1
	125×	29.19	31.00	31.25	0.00	131.6
	625×	**35.92**	**34.63**	34.34	0.55	22.3
	3,125×	37.22	36.81**^b^**	**37.10**	**2.78**	**5.2**
	15,625×	N	N	N	N	1.0**^a^**
	78,125×	N	N	N	N	N

Bold values means that the corresponding dilution is the highest dilution.

^a^Two of three replicates were tested positive;

^b^One of three replicates was tested positive. N represents three replicates negative;

^c^The final viral RNA copy number was defined as the higher value between the copy numbers of the two genes.

### Comparison of the 4 Methods in the Test of Clinical Samples

A total of 204 samples collected from 68 patients (all the 3 sample types were collected from each patient) were tested. The results showed that 55.9% (114/204) of the samples were positive by at least one of the 4 methods. The positivity rate of three methods (RT-PCR B, RT-RAA and dd-RT-PCR) were higher than 90% (91.2%, 91.2% and 93.0%, respectively), while RT-PCR A showed the lowest positivity rate (71.9%, 82/114). The positivity rate of dd-RT-PCR was the highest in every sample type (83.3% in OPS, 97.6% in NPS, and 93.8% in saliva). If OPS samples were tested alone by RT-PCR A, only 21.4% (12/56) of the COVID-19 patients would be considered positive (**Tables 4** and **S1**).

**Table 4:** Performance of 4 methods in testing 204 samples from 68 patients.

Sample type (n)	Method	No. of positive sample	No. of positive sample by any method	Positivity rate^a^ [%(95% CI)]	No. of positive patients by any methods in any sample types	Positivity rate^b^ [% (95% CI)]
OPS (n=68)	qRT-PCR A	12	24	50.0 (29.6- 70.3)	56^c^	21.4 (12.0- 34.8)
	qRT-PCR B	18		75.0 (52.9- 89.4)		32.1 (20.6- 46.1)
	RT-RAA	20		83.3 (61.8- 94.5)		35.7 (23.7- 49.7)
	dd-RT-PCR	20		83.3 (61.8- 94.5)		35.7 (23.7- 49.7)
NPS (n=68)	qRT-PCR A	33	42	78.6 (62.8- 89.2)		58.9 (45.0- 71.6)
	qRT-PCR B	41		97.6 (85.9-99.9)		73.2 (59.5- 83.8)
	RT-RAA	39		92.9 (79.4- 98.1)		69.6 (55.7- 80.8)
	dd-RT-PCR	41		97.6 (85.9-99.9)		73.2 (59.5- 83.8)
Saliva (n=68)	qRT-PCR A	37	48	77.1 (62.3- 87.5)		66.1 (52.1- 77.8)
	qRT-PCR B	45		93.8 (81.8- 98.4)		80.4 (67.2- 89.3)
	RT-RAA	45		93.8 (81.8- 98.4)		80.4 (67.2- 89.3)
	dd-RT-PCR	45		93.8 (81.8- 98.4)		80.4 (67.2- 89.3)
Total (n=204)	qRT-PCR A	82	114	71.9 (62.6- 79.7)	/	/
	qRT-PCR B	104		91.2 (84.1- 95.5)	/	/
	RT-RAA	104		91.2 (84.1- 95.5)	/	/
	dd-RT-PCR	106		93.0 (86.2- 96.7)	/	/

^a^No. of positive sample/No. of positive sample by any method.

^b^No. of positive sample/No. of positive patients by any methods in any sample types.

^c^The remaining 12 confirmed patients were negative by any methods in any sample types in sampling day.

In addition, the performance of the 4 methods in testing 197 saliva samples from 12 patients who were sampled almost daily since admission were also compared. Among these samples, 166 (84.3%) were SARS-CoV-2-positive by at least one of the 4 methods. The positivity rate of RT-PCR A, RT-PCR B, RT-RAA, and dd-RT-PCR was 86.7%, 91.0%, 91.0% and 94.6%, respectively (**Tables 5** and **S2**).

**Table 5:** Performance characteristics of 4 methods in testing 197 saliva samples from 12 patients.

Method	No. of positive samples	No. of positive samples by any method	Positivity rate^a^ (% [95% CI])
RT-PCR A	144	166	86.7 (80.4- 91.3)
RT-PCR B	151		91.0 (85.2- 94.7)
RT-RAA	151		91.0 (85.2- 94.7)
dd-RT-PCR	157		94.6 (89.6- 97.3)

^a^Number of positive samples divided by the number of positive samples by any method.

### Effect of Sample Type on Detection Accuracy

To determine the best sample type or combination of sample types in minimizing false negatives in the identification of SARS-CoV-2 infection, we analyzed and compared the number of positive results detected by different methods using different sample types and sample combinations. Regardless of the method used, the positivity rate in testing saliva samples (80.4%~86.5%) was always the highest, followed NPS (71.7%~78.8%) and OPS (26.1%~38.5%). In contrast, the accuracy of the test can be improved to nearly 100% for each method when saliva and NPS from the same patient were tested simultaneously (**Table 6**).

**Table 6:** Number of positive results detected by different methods using different sample types and sample combinations.

Sample types or combinations	No. of positive sample types or combinations, n (%^a^)				
	RT-PCR A	RT-PCR B	RT-RAA	dd-RT-PCR	any method^b^
OPS	12 (26.1)	18 (34.6)	20 (38.5)	20 (37.7)	24 (42.9)
NPS	33 (71.7)	41 (78.8)	39 (75.0)	41 (77.4)	42 (75.0)
Saliva	37 (80.4)	45 (86.5)	45 (86.5)	45 (84.9)	48 (85.7)
OPS + NPS^c^	34 (73.9)	41 (78.8)	41 (78.8)	43 (81.1)	46 (82.1)
OPS+ Saliva^c^	37 (80.4)	45 (86.5)	45 (86.5)	46 (86.8)	49 (87.5)
NPS+ Saliva^c^	46 (100)	52 (100)	52 (100)	52 (98.1)	55 (98.2)
OPS+ NPS+ Saliva^c^	46 (100)	52 (100)	52 (100)	53 (100)	56 (100)

^a^The number of positive samples of each sample type or sample combination divided by the total number of positive samples.

^b^Number of positive samples by any method.

^c^The combined sample was considered positive if any of the two or three samples from the same patient was positive.

**Figure 2:**
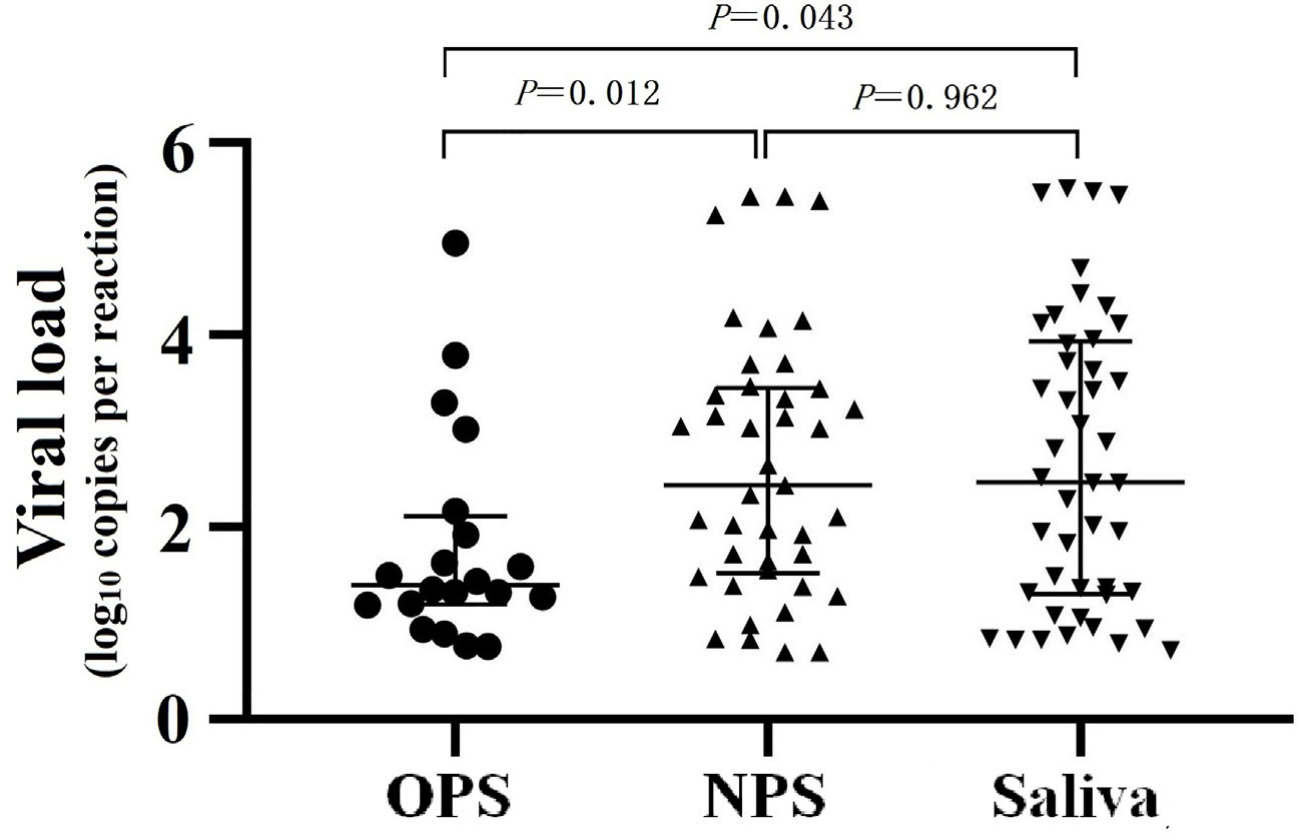
SARS-CoV-2 viral load in different sample types detected by dd-RT-PCR.

We further compared the absolute viral load in the 3 sample types of the 68 patients by dd-RT-PCR, and found that the median viral load of OPS (24.90 copies/test (IQR 15.58-129.85)) was significantly lower than that of NPS (274.40 copies/test (IQR 33.10-2836.45) and saliva (292.30 copies/test (IQR 20.20-8628.55) (**Figure 2**).

### Performance of the 4 Methods for Repeatedly Tested Patients

**Figure 3:**
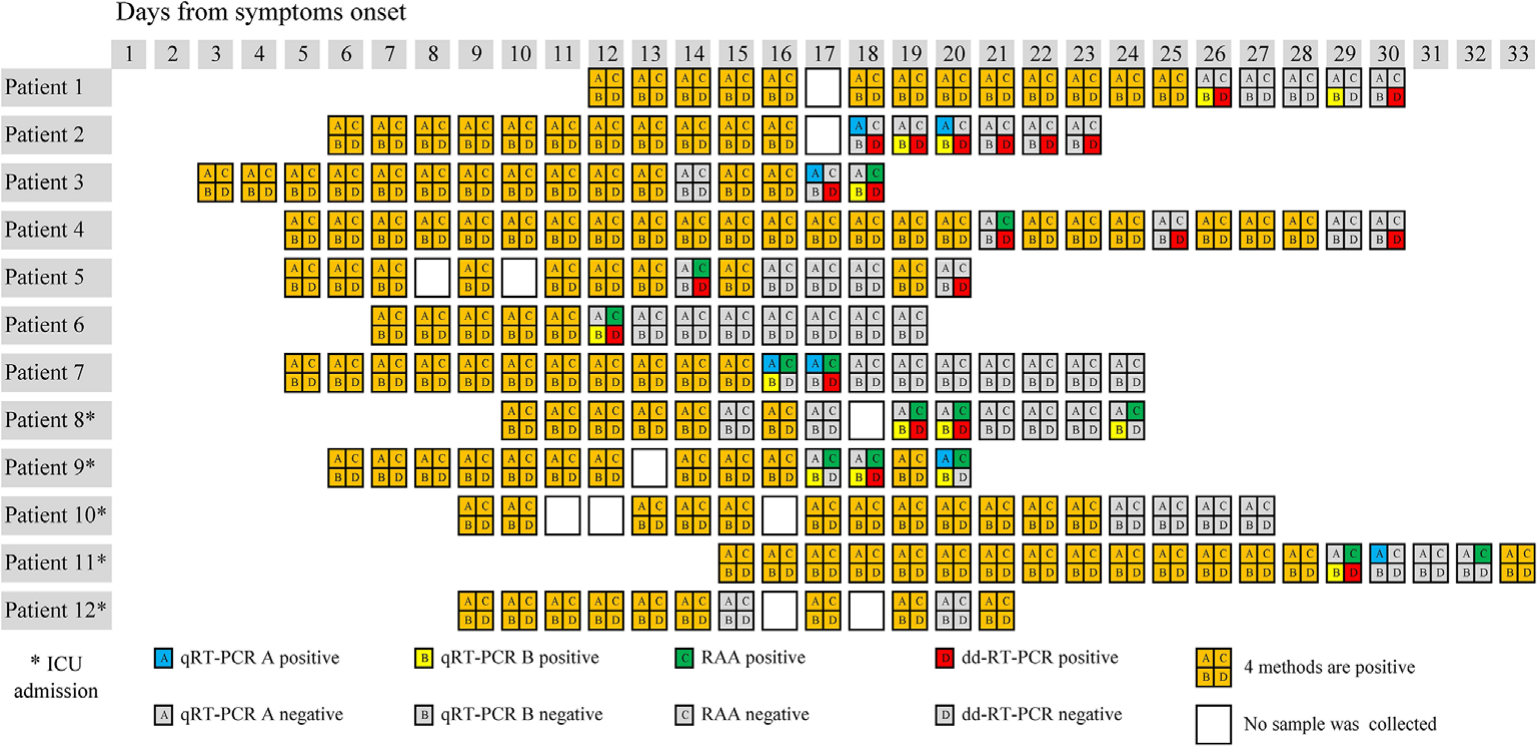
Results of the 4 methods in the testing of 197 saliva samples of 12 patients after admission.

Saliva samples from 12 patients who were sampled almost daily since admission were enrolled to dynamically evaluate the performance of the 4 methods. Among the 12 patients, samples of 5 patients were collected every day, while samples of 7 patients were missing on 1-2 days after onset. These patients were admitted to the hospital 3~14 days after the onset of the disease (**Figure 3**). We found that all the results were positive for the samples collected from most of the 12 patients within the first 1~2 weeks after admission using four methods. During the later stages of the disease, the results of different methods varied. Only dd-RT-PCR produced positive results for the samples collected on days 21, 22, and 23 from patient 2.

## Discussion

Nucleic acid testing of SARS-CoV-2 is a vital basis for confirming the diagnosis of suspected COVID-19 patients and determining whether they should be quarantined or can be discharged. Currently, although various respiratory specimen types can be used for testing in the case of respiratory infections such as influenza and SARS-CoV-2, the positive rate of different sample types is different (Kim et al., 2017; To et al., 2019; Lin et al., 2020; Wang W. et al., 2020). And the false negative results of nucleic acid tests in clinical specimens have restricted rapid and accurate diagnosis. In many cases, repeated sampling must be required, combined with testing of different types of samples and clinical examination methods (such as computed tomography), to confirm the cases (Ai et al., 2020). Additionally, a large number of testing reagents are urgently needed to meet the needs of clinical screening and diagnosis, leading to the emergency usage of multiple newly developed methods which rarely have been tested before. Thus, selection of the most appropriate respiratory sample type, method and sample combination that affect the accuracy of nucleic acid detection are needed. And the clinical detection performance of these test kits or methods also needs to be objectively evaluated.

Our research showed that the detection abilities of different detection methods differed. The method with the highest positivity rate was dd-RT-PCR, followed by RT-RAA and RT-PCR B, whereas RT-PCR A performed the worst. The dd-RT-PCR method had the advantage of absolute quantification without need of the standards when compared to other RT-PCR methods, as well as no amplification bias caused by interfering substances in the samples (Hindson et al., 2011; Li et al., 2018). Suo et al. also found that the sensitivity was improved from 40% for RT-PCR to 94% for dd-RT-PCR in SARS-CoV-2 detection on throat swab of 63 suspected patients and 14 supposed convalescents (Suo et al., 2020). According to our results, dd-RT-PCR performed better in detecting low-viral-load samples in the late stage of disease and reduced the false negative reports, which could be a powerful complement to the RT-PCR. RT-RAA is simple and time-saving with credible sensitivity and specificity. Furthermore, minimal requirements for equipment and resources make it especially suitable for on-site inspection in the underdeveloped areas, which has broad application prospects (Li et al., 2020). It was reported that the established POCT assay-based RAA offered 100% specificity and 100% sensitivity in the detection of clinical respiratory tract samples from COVID-19 patients when compared with RT-PCR (Xue et al., 2020; Zheng et al., 2021). Additionally, a multiple-center clinical evaluation of RT-RAA kit using respiratory tract samples (throat swabs, sputum, nasopharyngeal swabs, nasal swabs and bronchoalveolar lavage fluid) and non-respiratory samples (stool and whole blood) show that the total coincidence rate was 97.78% and the kappa value 0.952 (p < 0.05) compared to the commercial RT-PCR kits (Wang J. et al., 2020). In the present study, the positivity rate of RT-RAA was second only to dd-RT-PCR and nearly the same as RT-PCR B. It is concluded that RT-RAA is a reliable method worthy of promotion. RT-PCR is the most widely used method for respiratory virus detection. In our study, the positivity rate of RT-PCR A was lower than that of RT-PCR B, even though RT-PCR A is a two-gene (ORF1ab and N) testing, while RT-PCR B only tests one gene (ORF1ab). Primer design may be one of the problems, which is the most common cause of false negatives in RT-PCR (Jung et al., 2020). Therefore, to minimize false negatives, laboratories should choose the nucleic acid detection method with the higher positivity rate according to the laboratory conditions.

In our study, four methods were used for simultaneous testing, and the gold standard for diagnosis was any positive result of the four methods. The results demonstrated that saliva specimens showed higher positivity rate than NPS and OPS and supported the findings of previous studies (Fan et al., 2020; To et al., 2020; Sakanashi et al., 2021). It may be attributed to high expression of ACE2 in the alveoli, which is the receptor of SARS-CoV-2, making a large amount of virus accumulate in the lower respiratory tract (Fan et al., 2020; Wan et al., 2020). After a deep cough, the virus is flushed out with air pressure and wrapped in sputum or saliva, thereby making saliva to contain a higher viral load and easier to be detected positive (Fan et al., 2020; Wan et al., 2020). A recent study in Hong Kong found that the median load of SARS-CoV-2 in saliva can reach 3.3 × 10^6^ copies/mL (range from 9.9 × 10^2^ to 1.2 × 10^8^ copies/mL) (To et al., 2020). Some other studies have shown that the concentration of SARS-CoV-2 in NPS is higher than that in OPS (Zou et al., 2020). However, the collection of NPS is relatively complicated and causes significant discomfort to patients and is associated with infection risk to healthcare workers (Frazee et al., 2018; Ai et al., 2020). Due to the fast and convenient collection of OPS, it has become the first choice of many clinics. Consistent with some studies (Fan et al., 2020; Lin et al., 2020), the detection rate of OPS was as low as 42.9% in our study. And the absolute quantitative results of dd-RT-PCR also showed that the viral load in OPS was much lower than that in other type of samples, indicating a high rate of missed detection when testing samples with low viral loads. Besides, we also found that about four-fifths of the positive COVID-19 patients would be missed when detecting positive patients’ OPS using RT-PCR A, which raised serious risk for SARS-CoV-2 transmission. Therefore, it is prompted that use of inappropriate sample types was the most important reason for missed detection. According to our results, saliva after deep cough can be collected by patients themselves in a noninvasive manner, which is suitable for nucleic acid detection of SARS-CoV-2. The sample collection principle is to wear a mask and deep cough 3 to 5 times, and then immediately open the sterile container behind the mask and spit out saliva. There are several advantages of using saliva after deep cough for clinical detection, including better patient compliance, simple operation, reduced risk of medical staff infection and overcoming the shortage of personal protective equipment and specimen sampling tools. However, it should be noted that false negatives may still occur even when using saliva samples. As shown in **Figure 2**, a negative result suddenly appeared one day during a patient’s continued positive phase, such as patient 3 on day 14, patient 8 on day 15, and patient 12 on day 15. According to these results, we found that supplemental NPS testing can make up for missed detection of saliva samples. Taken together, for highly suspected or confirmed patients before discharging from the hospital, saliva after deep cough can be collected as well as NPS for simultaneous testing. We also recommend adding the evaluation of saliva during registration evaluation of SARS-CoV-2 nucleic acid detection reagents.

We acknowledge our limitations. Firstly, all patients enrolled in our study are confirmed COVID-19 patients, while samples of non-confirmed patients or patients with other respiratory tract pathogenic infections were not included. And the sample was considered true positive if it was positive detected by any method. Thus, it is difficult to analyze the sensitivity and specificity of different methodologies in this study. Secondly, given that COVID-19 patients mostly have dry cough with less sputum, we require that all patients wear masks and deep cough 3~5 times before spitting saliva into a sterile container when sampled. We called the sample “saliva after deep cough”. By this new sampling method, almost all patients are able to collect this specimen by themselves, except some patients who are unconscious. Thus, it was indeed difficult to distinguish sputum from saliva samples. Finally, this study was a single center cohort study and only samples from hospitalized patients were enrolled, which could lead to an unbalanced distribution of confounders when evaluating the positivity rate of different methods.

In conclusion, saliva after deep cough is preferred for the detection of SARS-CoV-2 and NPS should be collected for simultaneous testing if necessary. It is not recommended to only collect OPS for SARS-CoV-2 detection. Furthermore, laboratories should fully evaluate the nucleic acid detection methods, and highly sensitive methods like dd-RT-PCR are recommended to reduce missed diagnosis.

## Data Availability Statement

The raw data supporting the conclusions of this article will be made available by the authors, without undue reservation.

## Ethics Statement

The studies involving human participants were reviewed and approved by Clinical Research Ethics Committee of the First Affiliated Hospital, College of Medicine, Zhejiang University. Written informed consent for participation was not required for this study in accordance with the national legislation and the institutional requirements.

## Author Contributions

YC, BL, and FY designed the study. GX, FY, DZ, BF, QW, QZ, RW, XY and WC performed the experiments. FY, GX, SZ and DH analyzed the data. FY, GX, SZ, DH and JB wrote the manuscript. All authors contributed to the article and approved the submitted version.

## Funding

This work was supported by the National Natural Science Foundation of China (grant numbers 81803290, 81971919 and 82072377), National Key R&D Program of China (2020YFC0847800 and 2020YFC0848000) and Department of Education of Zhejiang Province (No. Y202043387).

## Conflict of Interest

The authors declare that the research was conducted in the absence of any commercial or financial relationships that could be construed as a potential conflict of interest.
