# Supplementary material for: Assessment of the Diagnostic Ability of Four Detection Methods Using Three Sample Types of COVID-19 Patients
Source: Front Cell Infect Microbiol. 2021 Jun 7;11:685640. doi: 10.3389/fcimb.2021.685640 (PMC8216554; doi:10.3389/fcimb.2021.685640)
Supplement: Supplementary file 1 [file DataSheet_1.docx]

## Supplementary materials

**Table S1 The calculation of test performance characteristics of 4 methods in testing 204 samples from 68 patients.**

|  | | 4 methods tested diagnosis | |  |
| --- | --- | --- | --- | --- |
|  |  | + ^a^ | - |  |
| qRT-PCR A | + | 82 | 0 | Report positive rate =  82/(82+32)*100% = 71.9% |
|  | - | 32 | 90 |  |
|  |  |  |  |  |
|  | | 4 methods tested diagnosis | |  |
|  |  | + ^a^ | - |  |
| qRT-PCR B | + | 104 | 0 | Report positive rate =  104/(10+104)*100% = 91.2% |
|  | - | 10 | 90 |  |
|  |  |  |  |  |
|  | | 4 methods tested diagnosis | |  |
|  |  | +^a^ | - |  |
| RT-RAA | + | 104 | 0 | Report positive rate =  104/(10+104)*100% = 91.2% |
|  | - | 10 | 90 |  |
|  |  |  |  |  |
|  | | 4 methods tested diagnosis | |  |
|  |  | +^a^ | - |  |
| dd-RT-PCR | + | 106 | 0 | Report positive rate =  106/(106+8)*100% = 93.0% |
|  | - | 8 | 90 |  |

^a^ Positive result in any of the four methods.

**Table S2 The calculation of test performance characteristics of 4 methods in testing 197 saliva samples from 12 patients.**

|  | | 4 methods tested diagnosis | |  |
| --- | --- | --- | --- | --- |
|  |  | +^a^ | - |  |
| qRT-PCR A | + | 144 | 0 | Report positive rate =  144/(144+22)*100% = 86.7% |
|  | - | 22 | 31 |  |
|  |  |  |  |  |
|  | | 4 methods tested diagnosis | |  |
|  |  | +^a^ | - |  |
| qRT-PCR B | + | 151 | 0 | Report positive rate =  151/(151+15)*100% = 91.0% |
|  | - | 15 | 31 |  |
|  |  |  |  |  |
|  | | 4 methods tested diagnosis ^a^ | |  |
|  |  | +^a^ | - |  |
| RT-RAA | + | 151 | 0 | Report positive rate =  151/(151+15)*100% = 91.0% |
|  | - | 15 | 31 |  |
|  |  |  |  |  |
|  | | 4 methods tested diagnosis ^a^ | |  |
|  |  | +^a^ | - |  |
| dd-RT-PCR | + | 157 | 0 | Report positive rate =  157/(157+9)*100% = 94.6 % |
|  | - | 9 | 31 |  |

^a^ Positive result in any of the four methods.
